# Circuiar of Information for Persens Seeking Appointment as Contract Surgeons in the U. S. Army

**Published:** 1899-01

**Authors:** 


### Circuiar of Information for Persens Seeking Appointment as Contract Surgeons in the U. S. Army.

---

Surgeon Kilbourne has issued the following circular:

The following requirements are exacted by the board of examiners: Evidence of graduation at a regular reputable medical college, diploma to be submitted to the board. Proof of hospital or other professional experience will be of benefit to the candidate. Candidates must be in good health, of reasonably sound physique, and citizens of the United States. The examination is of a practical nature, embracing hygiene, practice of medicine, pathology, and surgery. In addition a thesis on some professional topic will be prepared by the candidate. The following questions submitted to former candidates are published as a guide to applicants:

1. What chemical and physical qualities of water would lead you to suspect its potability?
2. What are the varieties and pathology of felon?
3. What is Widal's test?
4. What are the modern methods of treating diphtheria?

The pay of acting assistant surgeons is a hundred and fifty dollars monthly. The applicant must be free from physical defects which would incapacitate him from the military service.

For further information address Major H. S. Kilbourne, surgeon, United States Army, Army Building, 39 Whitehall Street, New York City, president of the board of examiners.

